# Modeling Markov Switching ARMA-GARCH Neural Networks Models and an Application to Forecasting Stock Returns

**DOI:** 10.1155/2014/497941

**Published:** 2014-04-06

**Authors:** Melike Bildirici, Özgür Ersin

**Affiliations:** ^1^Yıldız Technical University, Department of Economics, Barbaros Bulvari, Besiktas, 34349 Istanbul, Turkey; ^2^Beykent University, Department of Economics, Ayazağa, Şişli, 34396 Istanbul, Turkey

## Abstract

The study has two aims. The first aim is to propose a family of nonlinear GARCH models that incorporate fractional integration and asymmetric power properties to MS-GARCH processes. The second purpose of the study is to augment the MS-GARCH type models with artificial neural networks to benefit from the universal approximation properties to achieve improved forecasting accuracy. Therefore, the proposed Markov-switching MS-ARMA-FIGARCH, APGARCH, and FIAPGARCH processes are further augmented with MLP, Recurrent NN, and Hybrid NN type neural networks. The MS-ARMA-GARCH family and MS-ARMA-GARCH-NN family are utilized for modeling the daily stock returns in an emerging market, the Istanbul Stock Index (ISE100). Forecast accuracy is evaluated in terms of MAE, MSE, and RMSE error criteria and Diebold-Mariano equal forecast accuracy tests. The results suggest that the fractionally integrated and asymmetric power counterparts of Gray's MS-GARCH model provided promising results, while the best results are obtained for their neural network based counterparts. Further, among the models analyzed, the models based on the Hybrid-MLP and Recurrent-NN, the MS-ARMA-FIAPGARCH-HybridMLP, and MS-ARMA-FIAPGARCH-RNN provided the best forecast performances over the baseline single regime GARCH models and further, over the Gray's MS-GARCH model. Therefore, the models are promising for various economic applications.

## 1. Introduction

In the light of the significant improvements in the econometric techniques and in the computer technologies, modeling the financial time series have been subject to accelerated empirical investigation in the literature. Accordingly, following the developments in the nonlinear techniques, analyses focusing on the volatility in financial returns and economic variables are observed to provide significant contributions. It could be stated that important steps have been taken in terms of nonlinear measurement techniques focusing on the instability or stability occurring vis-a-vis encountered volatility. Further, the determination of stability or instability in terms of volatility in the financial markets gains importance especially for analyzing the risk encountered. In addition to impact of the magnitude and the size of shocks on volatility, the financial returns are under the influence of sudden or abrupt changes in the economy. Hence, the volatility of economic data has been explored in econometric literature as a result of the need of modelling uncertainty and risk in the financial returns. The relationship between the financial returns and various important factors such as the trade volume, market price of financial assets, and the relationship between volatility, trade volume, and financial returns have been vigorously investigated [1–4].

The ARCH model introduced by Engle [5] and the Generalized ARCH (GARCH) model introduced by Bollerslev [6] are generally accepted for measuring volatility in financial models. GARCH models have been used intensively in academic studies. A tremendous amount of GARCH models exist and various studies provide extended evaluation of the development.

Among many, Engle and Bollerslev [7] developed the Integrated-GARCH (I-GARCH) process to incorporate integration properties, AGARCH model, introduced by Engle [8], allows modeling asymmetric effects of negative and positive innovations. In terms of modeling asymmetries, GARCH models have been further developed by including asymmetric impacts of the positive and negative shocks to capture the asymmetric effects of shocks on volatility and return series which depend on the type of shocks, i.e. either negative or positive. Following the generalization of EGARCH model of Nelson [9] that allows modelling the asymmetries in the relationship between return and volatility, the Glosten et al. [10] noted the importance of asymmetry caused by good and bad news in volatile series and proposed a model that incorporates the past negative and positive innovations with an identity function that leads the conditional variance to follow different processes due to asymmetry. The finding is a result of the empirical analyses which pointed at the fact that the negative shocks had a larger impact on volatility. Consequently, the bad news have a larger impact compared to the conditional volatility dynamics followed after the good news. Due to this effect, asymmetric GARCH models have rapidly expanded. The GJR-GARCH model was developed independently by Zakoian [11, 12] and Glosten et al. [10]. It should be noted that, in terms of asymmetry, the Threshold GARCH (T-GARCH) of Zakoian [12], VGARCH, and nonlinear asymmetric GARCH models (NAGARCH) of Engle and Ng [13] are closely related versions to model asymmetry in financial asset returns. The SQR-GARCH model of Heston and Nandi [14] and the Aug-GARCH model developed by Duan [15] nest several versions of the models taking asymmetry discussed above. Further, models such as the Generalized Quadratic GARCH (GQARCH) model of Sentana [16] utilize multiplicative error terms to capture volatility more effectively. The FIGARCH model of Baillie et al. [17] benefits from an ARFIMA type fractional integration representation to better capture the long-run dynamics in the conditional variance (See for detailed information, Bollerslev [18]). The APARCH/APGARCH model of Ding et al. [19] is an asymmetric model that incorporates asymmetric power terms which are allowed to be estimated directly from the data. The APGARCH model also nests several models such as the TGARCH, TSGARCH, GJR, and logGARCH. The FIAPGARCH model of Tse [20] combines the FIGARCH and the APGARCH. Hyperbolic GARCH (HYGARCH) model of Davidson [21] nests the ARCH, GARCH, IGARCH, and FIGARCH models (for an extended review GARCH models, see Bollerslev [18]).

Even though the ARCH/GARCH models can be applied quickly for many time series, the shortcomings in these models were discussed by certain studies. Perez-Quiros and Timmermann [22] focused on the conditional distributions of financial returns and showed that recessionary and expansionary periods possess different characteristics, while the parameters of a GARCH model are assumed to be stable for the whole period. Certain studies discussed the high volatility persistence inherited in the baseline GARCH and proposed early signs of regime switches. Diebold [23] and Lamoureux and Lastrapes [24] are two of the highly cited studies discussing high persistence in volatility due to structural changes. Lamoureux and Lastrapes [24] showed that the encountered high persistence in volatility processes resulted from the volume effects that had not been taken into account. Qiao and Wong [25] followed a bivariate approach and confirmed that the Lamoureux and Lastrapes [24] effect exists due to the volume and turnover effects on conditional volatility and after the introduction of volume/turnover as exogenous variables, it is possible to obtain a significant decline in the the persistence. Mikosch and Stǎricǎ [26] showed that structural changes had an important impact that leads to accepting an integrated GARCH process. Bauwens et al. [27, 28] discussed that the persistence in the estimated single regime GARCH processes could be considered as resulting from the misspecification which could be controlled by introducing an MS-GARCH specification where the regime switches are governed by a hidden Markov chain.

Krämer [29] evaluated the autocorrelation in the squared error terms and provided an important contribution. Accordingly, the observed empirical autocorrelations of the *ε*_*t*_^2^ are much larger than the theoretical autocorrelations implied by the estimated parameters through evaluating an MS-GARCH model where the autocorrelation problem could be shown to accelerate as the transition probabilities approached 1. (For a proof see e.g., Francq and Zakoïan [30]; Krämer [29]) (In particular, the empirical autocorrelations of the *ε*_*t*_^2^ often seem to indicate long memory, which is not possible in the GARCH-model; in fact, in all standard GARCH-models, theoretical autocorrelations must eventually decrease exponentially, so long memory is ruled out). Alexander and Lazaar [31] showed that leverage effects are due to asymmetry in the volatility responses to the price shocks and the leverage effect accelerates once the markets are in the more volatile regime Krämer and Tameze [32] showed that a single state GARCH model had only one mean reversion while by allowing regime switching in the GARCH processes, mean reverting effect diminishes. In a perspective of volatility, if these shifts are persistent, then there are two sources of volatility persistence, due to shocks and due to regime-switching in the parameters of the variance process. By utilizing a Markov transformation model, it could be shown that the relationships among the regimes between the periods of *t* − 1 and *t* could be explained and the most important advantage of the MSGARCH model exposes itself as there is no need for the researchers to observe the regime changes. The model allows different regimes to reveal by itself [33].

The regime switching in light of the Markov switching model has interesting properties to be examined such as the stationarity by allowing the switching course of volatility inherent in the asset prices. The hidden Markov model (HMM) developed by Taylor [34] is a switching model that benefits from including an unobserved variable to capture volatility to be modeled with transitions between the hidden states that possess different probability distributions attached to each state. Hidden Markov model has been applied successfully by Alexander and Dimitriu [35], Cheung and Erlandsson [36], Francis and Owyang [37], and by Clarida et al. [38] to capture the switching type of predictions in stock returns, interest rates, and exchange rates. Regime switching model has been used extensively for prediction of returns belonging to different stock market returns in different economies and by following the fact that the stock market indices are very sensitive to stock volatility, which accelerates especially during periods with market turbulences (see for detailed information, Alexander and Kaeck, [39]).

The conventional statistical techniques for forecasting reached their limit in applications with nonlinearities, furthermore, recent results suggest that nonlinear models tend to perform better in models for stock returns forecasting [40]. For this reason, many researchers have used artificial neural networks methodologies for financial analysis on the stock market. Lai and Wong [41] contributed to the nonlinear time series modeling methodology by making use of single-layer neural network. Further, modeling of NN models for estimation and prediction for time series have important contributions. Weigend et al. [42], Weigend and Gershenfeld [43], White [44], Hutchinson et al. [45], and Refenes et al. [46] contributed to financial analyses, stock market returns estimation, pattern recognition, and optimization. NN modeling methodology is applied successfully by Wang et al. [47] and Wang [48] to forecast the value of stock indices. Similarly, Abhyankar et al. [49], Castiglione [50], Freisleben [51], Kim and Chun [52], Liu and Yao [53], Phua et al. [54], Refenes et al. [55], Resta [56], R. Sitte and J. Sitte [57], Tiňo et al. [58], Yao and Poh [59], and Yao and Tan [60] are important investigations focusing on the relationships between stock prices and market volumes and volatility. For similar applications, see [1–4]. Bildirici and Ersin [61] modeled NN-GARCH family models to forecast daily stock returns for short and long run horizons and they showed that GARCH models augmented with artificial neural networks (ANN) architectures and algorithms provided significant forecasting performances. Ou and Wang [62] extended the NN-GARCH models to Support Vector Machines. Azadeh et al. [63] evaluated NN-GARCH models and proposed the integrated ANN models. Bahrammirzaee [64] provided an analysis based on financial markets to evaluate the artificial neural networks, expert systems, and hybrid intelligence systems. Further, Kanas and Yannopoulos [65] and Kanas [40] used Markov switching and Neural Networks techniques for forecasting stock returns; however, their approaches depart from the approach followed within this study.

In this study, the neural networks and Markov switching structures are aimed to be integrated to augment the ARMA-GARCH models by incorporating regime switching and different neural networks structures. The approach aims at formulations and estimations of MS-ARMA-GARCH-MLP, MS-ARMA-APGARCH-MLP, MS-ARMA-FIGARCH-MLP, MS-ARMA-FIAPGARCH-MLP, MS-ARMA-GARCH-RBF MS-ARMA-APGARCH-RBF, MS-ARMA-FIGARCH-RBF, and MS-ARMA-FIAPGARCH-RBF; the recurrent neural network augmentations of the models are, namely, the MS-ARMA-GARCH-RNN MS-ARMA-APGARCH-RNN, MS-ARMA-FIGARCH-RNN, and MS-ARMA-FIAPGARCH-RNN. And lastly, the paper aims at providing Hybrid NN versions: the MS-ARMA-GARCH-HybridNN, MS-ARMA-APGARCH-HybridNN, MS-ARMA-FIGARCH-HybridNN, and MS-ARMA-FIAPGARCH-HybridNN.

## 2. The MS-GARCH Models

Over long periods, there are many reasons why financial series exhibit important breaks in behavior; examples include depression, recession, bankruptcies, natural disasters, and market panics, as well as changes in government policies, investor expectations, or the political instability resulting from regime change.

Diebold [23] provided a throughout analysis on volatility models. One of the important findings is the fact that volatility models that fail to adequately incorporate nonlinearity are subject to an upward bias in the parameter estimates which results in strong forms of persistence that occurs especially in high volatility periods in financial time series. As a result of the bias in the parameter estimates, one important result of this fact is on the out-of-sample forecasts of single regime type GARCH models. Accordingly, Schwert [66] proposed a model that incorporates regime switching that is governed by a two state Markov process, hence the model retains different characteristics in the regimes that are defined as high volatility and low volatility regimes.

Hamilton [67] proposed the early applications of HMC models within a Markov switching framework. Accordingly, MS models were estimated by maximum likelihood (ML) where the regime probabilities are obtained by the proposed Hamilton-filter [68–71]. ML estimation of the model is based on a version of the Expectation Maximization (EM) algorithm as discussed in Hamilton [72], Krolzig [73–76]. In the MS models, regime changes are unobserved and are a discrete state of a Markov chain which governs the endogenous switches between different AR processes throughout time. By inferring the probabilities of the unobserved regimes which are conditional on an information set, it is possible to reconstruct the regime switches [77].

Furthermore, certain studies aimed at the development of modeling techniques which incorporate both the probabilistic properties and the estimation of a Markov switching ARCH and GARCH models. A condition for the stationarity of a* natural *path-dependent Markov switching GARCH model as in Francq et al. [78] and a throughout analysis of the probabilistic structure of that model, with conditions for the existence of moments of any order, are developed and investigated in Francq and Zakoïan [30]. Wong and Li [79], Alexander and Lazaar [80], and Haas et al. [81–83] derived stationarity analysis for some mixing models of conditional heteroskedasticity [27, 28]. For the Markov switching GARCH models that avoid the dependency of the conditional variance on the chain's history, the stationarity conditions are known for some special cases in the literature [84]. Klaassen [85] developed the conditions for stationarity of the model as the special cases of the two regimes. A necessary and sufficient stationarity condition has been developed by Haas et al. [81–83] for their Markov switching GARCH model. Furthermore, Cai [86] showed the properties of Bayesian estimation of a Markov switching ARCH model where only the constant in the ARCH equation is allowed to have regime switches. The approach has been investigated by Kaufman and Frühwirth-Schnatter [87] and Kaufmann and Scheicher [88]. Das and Yoo [89] proposed an MCMC algorithm for the same model (switches being allowed in the constant term) with a single state GARCH term to show that gains could be achieved to overcome path-dependence. MS-GARCH models are studied by Francq and Zakoïan [30] to achieve their non-Bayesian estimation properties in light of the generalized method of moments. Bauwens et al. [27, 28] proposed a Bayesian Markov chain Monte Carlo (MCMC) algorithm that is differentiated by including the state variables in the parameter space to control the path-dependence by obtaining the parameter space with Gibbs sampling [90].

The high and low volatility probabilities of MS-GARCH models allow differentiating high and low volatility periods. By observing the periods in which volatility is high, it is possible to investigate the economic and political reasons that caused increased volatility. If a brief overview is to be presented, there are several models based on the idea of regime changes which should be mentioned. Schwert [66] explores a model in which switches between these states that returns can have a high or low variance are determined by a two-state Markov process. Lamoureux and Lastrapes [24] suggest the use of Markov switching models for a way of identifying the timing of the shifts in the unconditional variance. Hamilton and Susmel [91] and Cai [86] proposed Markov switching ARCH model to capture the effects of sudden shifts in the conditional variance. Further, Hamilton and Susmel [91] extended the analysis to a model that allows three regimes, which were differentiated between low, moderate and high volatility regimes, where the high-volatility regime captured the economic recessions. It is accepted that the proposals of Cai [86] and Hamilton and Susmel [91] helped the researchers to control for the problem of path dependence, which makes the computation of the likelihood function impossible (The conditional variance at time* t *depends on the entire sequence of regimes up to time* t *due to the recursive nature of the GARCH process. In Markov switching model, the regimes are unobservable, one needs to integrate over all possible regime paths. The number of possible paths grows exponentially with* t*, which renders ML estimation intractable.) (see for detail, Bauwens, et al. [27, 28]).

Gray [92] study is one of the important studies where a Markov switching GARCH model is proposed to overcome the path dependence problem. According to Gray's model, once the conditional volatility processes are differentiated between regimes, an aggregation of the conditional variances for the regimes could be used to construct a single variance coefficient to evaluate the path dependence. A modification is also conducted by Klaassen [85]. Yang [93], Yao and Attali [94], Yao [95], and Francq and Zakoïan [96] derived conditions for the asymptotic stationarity of some AR and ARMA models with Markov switching regimes. Haas et al. [81–83] investigated a MS-GARCH model by which a finite state-space Markov chain is assumed to govern the ARCH parameters, whereas the autoregressive process followed by the conditional variance is subject to the assumption that past conditional variances are in the same regime (for details, the readers are referred to Bauwens et al. [27, 28], Klaassen [85], Haas et al. [81–83], Francq and Zakoïan [30], Krämer [29], and Alexander and Kaeck [39]).

Another area of analysis pioneered by Haas [97] and Chang et al. [98] allow different distributions in order to gain forecast accuracy. An important finding of these studies showed that by allowing the regime densities to follow skew-normal distribution with Gaussian tail characteristics, several return series could be modeled more efficiently in terms of forecast accuracy. Liu [99] developed and discussed the conditions for stationarity in Markov switching GARCH structure in Haas et al. [81–83] and proved the existence of the moments. In addition, Abramson and Cohen [100] discussed and further evaluated the stationarity conditions in a Markov switching GARCH process and extended the analysis to a general case with* m*-state Markov chains and GARCH(*p*, *q*) processes. An evaluation and extension of the stationarity conditions for a class of nonlinear GARCH models are investigated in Abramson and Cohen [100]. Francq and Zakoïan [30] derived the conditions for weak stationarity and existence of moments of any order MS-GARCH model. Bauwens et al. [27, 28] showed that by enlarging the parameter space to include space variables, though maximum likelihood estimation is not feasible, the Bayesian estimation of the extended process is feasible for a model where the regime changes are governed with a hidden Markov chain. Further, Bauwens et al. [27, 28] accepted mild regularity conditions under which the Markov chain is geometrically ergodic and has finite moments and is strictly stationary.

### 2.1. MS-ARMA-GARCH Models

To avoid path-dependence problem, Gray [92] suggests integrating out the unobserved regime path in the GARCH term by using the conditional expectation of the past variance. Gray's MS-GARCH model is represented as follows:

(1) σt,(st)2=w(st)+∑i=1qαi,(st)εt−i2+∑j=1pβj,(st)E(εt−j2It−j−1)=w(st)+∑i=1qαi,(st)εt−i2 +∑j=1pβj,(st)∑st−j=1mP(st−j=st−jIt−j−1)σt−j2,st−j,

where *w*_*st*_ > 0, *α*_*i*,*st*_ ≥ 0, *β*_*j*,*st*_ ≥ 0, and *i* = 1,…, *q*, *j* = 1,…, *p*, *s*_*t*_ = 1,…, *m*. The probabilistic structure of the switching regime indicator *s*_*t*_ is defined as a first-order Markov process with constant transition probabilities *π*_1_ and *π*_2_, respectively (Pr{*s*_*t*_ = 1 | *s*_*t*−1_ = 1} = *π*_1_, Pr{*s*_*t*_ = 2 | *s*_*t*−1_ = 1} = 1 − *π*_1_, Pr{*s*_*t*_ = 2 | *s*_*t*−1_ = 2} = *π*_2_, and Pr{*s*_*t*_ = 1 | *s*_*t*−1_ = 2} = 1 − *π*_2_).

Although Dueker [101] accepts a collapsing procedure of Kim's [102] algorithm to overcome path-dependence problem, Dueker [101] adopts the same framework of Gray [92]. Accordingly, the modified GARCH version of Dueker [101] is accepted which governs the dispersion instead of traditional GARCH(1,1) specification.

Yang [103], Yao and Attali [94], Yao [95], and Francq and Zakoïan [96] derived conditions for the asymptotic stationarity of models with Markov switching regimes (see for detailed information Bauwens and Rombouts [104]. The major differences between Markov switching GARCH models are the specification of the* variance process*; that is, the conditional variance *σ*_*t*_^2^ = Var⁡(*ε*_*t*_/*S*_*t*_). To consider the conditional variance as in the Bollerslev's [105] GARCH model and to consider the regime dependent equation for the conditional variance in Frömmel [106] are accepted that The coefficients *w*_*st*_, *α*_*st*_, *β*_*st*_ correspond to respective coefficients in the one-regime GARCH model, but may differ depending on the present state.

Klaassen [85] (Klassen [85] model is defined as $\sigma_{t,(s_{t})}^{2}=w_{\left(s_{t}\right)}+\sum_{i=1}^{q}\alpha_{i,(s_{t})}\varepsilon_{t-i}^{2}+\sum_{j=1}^{p}\beta_{j,(s_{t})}\sum_{\tilde{s}=1}^{m}P\left(S_{t-j}=s_{t-j}|I_{t-1},S_{t}=s_{t}\right)\sigma_{t-j}^{2},s_{t-j}$) suggested to use the conditional expectation of the lagged conditional variance with a broader information set than the model derived in Gray [92]. Accordingly, Klaassen [85] suggested modifying Gray's [92] model by replacing *p*(*s*_*t*−*j*_ = *s*_*t*−*j*_ | *I*_*t*−*j*−1_)  by *p*(*s*_*t*−*j*_ = *s*_*t*−*j*_ | *I*_*t*−1_, *S*_*t*_ = *s*_*t*_) while evaluating  *σ*_*t*_^2^, *s*_*t*_.

Another version of MS-GARCH model is developed by Haas et al. [81–83]. According to this model, Markov chain controls the ARCH parameters at each regime (*w*_*s*_, *α*_*i*,*s*_) and the autoregressive behavior in each regime is subject to the assumption that the past conditional variances are in the same regime as that of the current conditional variance [100].

In this study, models will be derived following the MS-ARMA-GARCH specification in the spirit of Blazsek and Downarowicz [107] where the properties of MS-ARMA-GARCH processes were derived following Gray [92] and Klaassen [85] framework. Henneke et al. [108] developed an approach to investigate the model derived in Francq et al. [78] for which the Bayesian framework was derived. The stationarity of the model was evaluated by Francq and Zakoïan [96] and an algorithm to compute the Bayesian estimator of the regimes and parameters was developed. It should be noted that the MS-ARMA-GARCH models in this paper were developed by following the models developed in the spirit of Gray [92] and Klaassen [85] similar to the framework of Blazsek and Downarowicz [107].

The MS-ARMA-GARCH model with regime switching in the conditional mean and variance are defined as a regime switching model where the regime switches are governed by an unobserved Markov chain in the conditional mean and in the conditional variance processes as

(2) $$\begin{array}{c} y_{t}=c_{\left(s_{t}\right)}+\overset{r}{\underset{i=1}{\sum }}\theta_{i,(s_{t})}y_{t-i}+\varepsilon_{t,(s_{t})}+\overset{m}{\underset{j=1}{\sum }}\varphi_{j,(s_{t})}\varepsilon_{t-j,(s_{t})},\\ \sigma_{t,(s_{t})}^{2}=w_{\left(s_{t}\right)}+\overset{p}{\underset{i=1}{\sum }}\alpha_{i,(s_{t})}{\varepsilon^{2}}_{t-i,(s_{t})}+\overset{q}{\underset{j=1}{\sum }}\beta_{\left(s_{t}\right)}\sigma_{t-j,(s_{t})}, \end{array}$$

where,

(3) $$\begin{array}{c} \varepsilon_{t-i-1,(s_{t-i})}=E\left[\varepsilon_{t-i-1,(s_{t-i-1})}\;|\;s_{t-i},Y_{t-i-1}\right],\\ \sigma_{t-i-1,(s_{t-i})}=E\left[\varepsilon_{t-i-1,(s_{t-i-1})}\;|\;s_{t-i},Y_{t-i-1}\right]. \end{array}$$

Thus, the parameters have nonnegativity constraints *ϕ*, *θ*, *φ*, *w*, *α*, *β* > 0 and the regimes are determined by *s*_*t*_,

(4) $$\begin{array}{c} L=\overset{T}{\underset{t=1}{\prod }}f\left(y_{t}\;|\;s_{t}=i,Y_{t-1}\right)\text{Pr}\left[s_{t}=i\;|\;Y_{t-1}\right], \end{array}$$

and the probability Pr[*s*_*t*_ = *i* | *Y*_*t*−1_] is calculated through iteration:

(5) πjt=Pr[st=j ∣ Yt−1]=∑i=01Pr[st=j ∣ st−1=i]Pr[st=j ∣ Yt−1]∑i=01ηjiπit−1∗.

Accordingly, the two models, the Henneke et al. [108] and the Francq et al. [78] approaches, could be easily differentiated through the definitions of *ε*_*t*−1_^2^ and *σ*_*t*−1_. Further, asymmetric power terms and fractional integration will be introduced to the derived model in the following sections.

### 2.2. MS-ARMA-APGARCH Model

Liu [99] provided a generalization of the Markov switching GARCH model of Haas et al. [81–83] and derived the conditions for stationarity and for the existence of moments. Liu [99] proposes a model which allowed for a nonlinear relation between past shocks and future volatility as well as for the leverage effects. The leverage effect is an outcome of the observation that the reaction of stock market volatility differed significantly to the positive and the negative innovations. Haas et al. [109, 110] complements Liu's [99] work in two ways. Firstly, the representation of the model developed by Haas [109] allows computational ease for obtaining the unconditional moments. Secondly, the dynamic autocorrelation structure of the power-transformed absolute returns (residuals) was taken as a measure of volatility.

Haas [109] model assumes that time series {*ε*_*t*_, *t* ∈ **Z**} follows a* k* regime MS-APGARCH process,

(6) $$\begin{array}{c} \varepsilon_{t}=\eta_{t}\sigma_{\Delta_{t},t}\;t\in Z, \end{array}$$

with {*η*_*t*_, *t* ∈ **Z**} being* i.i.d.* sequence and {Δ_*t*_, *t* ∈ **Z**} is a Markov chain with finite state space *S* = {1,…, *k*} and *P* is the irreducible and aperiodic transition matrix with typical element *p*_*ij*_ = *p*(Δ_*t*_ = *j* | Δ_*t*−1_ = *i*) so that

(7) $$\begin{array}{c} P=\left[p_{ij}\right]=\left[p\left(\Delta_{t}=j\;|\;\Delta_{t-1}=i\right)\right],\;i,j=1,\ldots ,k. \end{array}$$

The stationary distribution of Markov-chain is shown as *π*_*∞*_ = (*π*_1,*∞*_,…,*π*_*k*,*∞*_)′.

According to the Liu [99] notation of MS-APGARCH model, the conditional variance *σ*_*jt*_^2^ of* j*th regime follows a univariate APGARCH process as follows:

(8) $$\begin{array}{c} \sigma_{jt}^{\delta }=w_{j}+\alpha_{1j}{\left|\varepsilon_{t-1}^{+}\right|}^{\delta }+\alpha_{2j}{\left|\varepsilon_{t-1}^{-}\right|}^{\delta }+\beta_{j}^{}\sigma_{j,t-1}^{\delta },\;\delta >0, \end{array}$$

where, *w*_*j*_ > 0, *α*_1*j*_, *α*_2*j*_, *β*_*j*_ ≥ 0, *j* = 1,…, *k*. For the power term *δ* = 2 and for *α*_1*j*_ = *α*_2*j*_, the model in (8) reduces to MS-GARCH model. Similar to the Ding et al. [19], the asymmetry, which is called “leverage effect,” is captured by *α*_1*j*_ ≠ *α*_2*j*_ [109]. If the past negative shocks have deeper impact, parameters are expected to be *α*_1*j*_ < *α*_2*j*_ so that the leverage effect becomes stronger.

Another approach that is similar to Liu [99] model is the Haas [109] model, where the asymmetry terms have a differentiated form as

(9) $$\begin{array}{c} \sigma_{jt}^{\delta }=w_{j}+\alpha_{j}{\left(\left|\varepsilon_{t-1}\right|-\gamma_{j}\varepsilon_{t-1}\right)}^{\delta }+\beta_{j}^{}\sigma_{j,t-1}^{\delta },\;\delta >0, \end{array}$$

with the restrictions 0 < *w*_*j*_, *α*_*j*_, *β*_*j*_ ≥ 0, *γ*_*j*_ ∈ [−1,1] with regimes *j* = 1,…, *k*. The MS-APGARCH model of Haas [109] reduces to Ding et al. [19] single regime APGARCH model if *j* = 1. Equation (9) reduces to Liu [99] MS-APGARCH specification if *α*_1*j*_ = *α*_*j*_(1−*γ*_*j*_)^*δ*^and *β*_*j*_ = *α*_*j*_^*s*^(1+*γ*_*j*_)^*δ*^.

The MS-ARMA-GARCH type model specification in this study assumes that the conditional mean follows MS-ARMA process, whereas the conditional variance follows regime switching in the GARCH architecture. Accordingly, MS-ARMA-APGARCH architecture nests several models by applying certain restrictions. The MS-ARMA-APGARCH model is derived by moving from MS-ARMA process in the conditional mean and MS-APGARCH(*l*, *m*) conditional variance process as follows:

(10) σt,(st)δ(st)=w(st)+∑l=1rαl,(st)(|εt−l|−γl,(st)εt−l)δ(st) +∑m=1qβm,(st)σt−m,(st)δ(st), δ(st)>0,

where the regime switches are governed by (*s*_*t*_) and the parameters are restricted as *w*_(*s*_*t*_)_ > 0, *α*_*l*,(*s*_*t*_)_, *β*_*m*,(*s*_*t*_)_ ≥ 0 with  *γ*_*l*,(*s*_*t*_)_ ∈ (−1, 1), *l* = 1,…, *r*. One important difference is that MS-ARMA-APGARCH model in (10) allows the power parameters to vary across regimes. Further, if the following restrictions are applied, *l* = 1, *j* = 1, *δ*_(*s*_*t*_)_ = *δ*, the model reduces to the model of Haas [109] given in (9).

In applied economics literature, it is shown that many financial time series possess long memory, which can be fractionally integrated. Fractional integration will be introduced to the MS-ARMA-APGARCH model given above.

### 2.3. MS-ARMA-FIAPGARCH Model 

Andersen and Bollerslev [111], Baillie et al. [17], Tse [112], and Ding et al. [19] provided interesting applications in which the attention had been directed on long memory. Long memory could be incorporated to the model above by introducing fractional integration in the conditional mean and the conditional variance processes.

MS-ARMA-FIAPGARCH derived is a fractional integration augmented model as follows:

(11) (1−β(st)L)σt,(st)δ(st) =ω+((1−β(st)L)−(1−α(st)L)(1−L)d(st))  ×(|εt−1|−γ(st)εt−1)δ(st),

where the lag operator is denoted by *L*, autoregressive parameters are *β*_(*s*_*t*_)_, and *α*_(*s*_*t*_)_ shows the moving average parameters, *δ*_(1)_ > 0 denotes the optimal power transformation, the fractional differentiation parameter varies between 0 ≤ *d*_(*s*_*t*_)_ ≤ 1 and allows long memory to be integrated to the model. Regime states (*s*_*t*_) are defined with *m* regimes as *i* = 1,…, *m*. The asymmetry term |*γ*_(*s*_*t*_)_| < 1 ensures that positive and negative innovations of the same size may have asymmetric effects on the conditional variance in different regimes.

Similar to the MS-ARMA-APGARCH model, the MS-ARMA-FIAPGARCH model nests several models. By applying *δ*_(*s*_*t*_)_ = 2, the model reduces to Markov switching fractionally integrated asymmetric GARCH (MS-ARMA-FIAGARCH); if *δ*_(*s*_*t*_)_ = 2 restriction is applied with *γ*_(*s*_*t*_)_ = 0, the model reduces to Markov switching FIGARCH (MS-ARMA-FIGARCH). For *d*_(*s*_*t*_)_ = 0, model reduces to the short memory version, the MS-ARMA-APGARCH model, if the additional constraint *δ*_(*s*_*t*_)_ = 2 is applied, the model reduces to MS-Asymmetric GARCH (MS-AGARCH). Lastly, for all the models mentioned above if *i* = 1, all models reduce to single regime versions of the relevant models, namely, the FIAPGARCH, FIAGARCH, FIGARCH, and AGARCH models, their relevant single regime variants. For a typical, with the constraints *i* = 1 and *δ*_(*s*_*t*_)_ = 2, *γ*_(*s*_*t*_)_ = 0, the model reduces to single regime FIGARCH model of Baillie et al. [17]. To differentiate between the GARCH specifications, forecast performance criteria comparisons are assumed.

## 3. Neural Network and MS-ARMA-GARCH Models

In this section of the study, the MultiLayer Perceptron, Radical Basis Function, and Recurrent Neural Network models that belong to the ANN family will be combined with Markov switching and GARCH models. In this respect, Spezia and Paroli [113] is another study that merged the Neural Network and MS-ARCH models.

### 3.1. Multilayer Perceptron (MLP) Models

#### 3.1.1. MS-ARMA-GARCH-MLP Model

Artificial Neural Network models have many applications in modeling of functional forms in various fields. In economics literature, the early studies such as Dutta and Shektar [114], Tom and Kiang [115], Do and Grudinsky [116], Freisleben [51], and Refenes et al. [55] utilize ANN models to option pricing, real estates, bond ratings, and prediction of banking failures among many, whereas studies such as Kanas [40], Kanas and Yannopoulos [65], and Shively [117] applied ANN models to stock return forecasting, and Donaldson and Kamstra [118] proposed hybrid modeling to combine GARCH, GJR, and EGARCH models with ANN architecture.

The MLP, an important class of neural networks, consists of a set of sensory units that constitute the input layer, one or more hidden layers, and an output layer. The additional linear input which is connected to the MLP network is called the Hybrid MLP. Hamilton model can also be considered as a nonlinear mixture of autoregressive functions, such as the multilayer perceptron and thus, the Hamilton model is called Hybrid MLP-HMC models [119]. Accordingly, in the HMC model, the regime changes are dominated by a Markov chain without making a priori assumptions in light of the number of regimes [119]. In fact, Hybrid MLP accepts the network inputs to be connected to the output nodes with weighted connections to form a linear model that is parallel with nonlinear Multilayer Perceptron.

In the study, the MS-ARMA-GARCH-MLP model to be proposed allows Markov switching type regime changes both in the conditional mean and conditional variance processes augmented with MLP type neural networks to achieve improvement in terms of in-sample and out-of-sample forecast accuracy.

The MS-ARMA-GARCH-MLP model is defined of the form:

(12) $$\begin{array}{c} y_{t}=c_{\left(s_{t}\right)}+\overset{r}{\underset{i=1}{\sum }}\theta_{i,(s_{t})}y_{t-i}+\varepsilon_{t,(s_{t})}+\overset{n}{\underset{j=1}{\sum }}\varphi_{j,(s_{t})}\varepsilon_{t-j,(s_{t})}, \end{array}$$

(13) σt,2(st)=w(st)+∑p=1pαp,(st)εt−p2,(st)+∑q=1qβq,(st)σt−q,(st) +∑h=1hξh,(st)ψ(τh,(st),Zt,(st)λh,(st),θh,(st)),

where, the regimes are governed by unobservable Markov process:

(14) ∑i=1mσt(i)2P(St=i ∣ zt−1), i=1,…m.

In the MLP type neural network, the logistic type sigmoid function is defined as

(15) ψ(τh,(st),Zt,(st)λh,(st),θh,(st))=[1+exp⁡(−τh,(st)(∑l=1l[∑h=1hλh,l,(st)zt−l,(st)h+θh,(st)]))]−1

(16) $$\begin{array}{c} \left(\frac{1}{2}\right)\lambda_{h,d}\sim \;\text{uniform}\;\;\left[-1,+1\right] \end{array}$$

and *P*(*S*_*t*_ = *i* | *z*_*t*−1_), the filtered probability with the following representation,

(17) $$\begin{array}{c} \left(P\left(S_{t}=i\;|\;z_{t-1}^{}\right)\alpha f\left(P\left(\sigma_{t-1}\;|\;z_{t-1}^{},s_{t-1}=1\right)\right)\right) \end{array}$$

if *n*_*j*,*i*_ transition probability *P*(*s*_*t*_ = *i* | *s*_*t*−1_ = *j*) is accepted;

(18) $$\begin{array}{c} z_{t-d}=\frac{\left[\varepsilon_{t-d}-E\left(\varepsilon \right)\right]}{\sqrt{E\left(\varepsilon^{2}\right)}} \end{array}$$

*s* → max⁡{*p*, *q*} recursive procedure is started by constructing *P*(*z*_*s*_ = *i* | *z*_*s*−1_), where *ψ*(*z*_*t*_ *λ*_*h*_) is of the form 1/(1 + exp⁡(−*x*)), a twice-differentiable, continuous function bounded between [0, 1]. The weight vector *ξ* = *w*; *ψ* = *g* logistic activation function and input variables are defined as *z*_*t*_ *λ*_*h*_ = *x*_*i*_, where *λ*_*h*_ is defined as in (16).

If *n*_*j*,*i*_ transition probability *P*(*z*_*t*_ = *i* | *z*_*t*−1_ = *j*) is accepted,

(19) f(yt ∣ xt,zt=i)=12πht(i)exp⁡{−(yt−xt′φ−∑j=1Hβjp(xt′γj))22ht(j)},

*s* → max⁡{*p*, *q*}, recursive procedure is started by constructing *P*(*z*_*s*_ = *i* | *z*_*s*−1_).

#### 3.1.2. MS-ARMA-APGARCH-MLP Model

Asymmetric power GARCH (APGARCH) model has interesting features. In the construction of the model, the APGARCH structure of Ding et al. [19] is followed. The model given in (13) is modified to obtain the Markov switching APGARCH Multilayer Perceptron (MS-ARMA-APGARCH-MLP) model of the form,

(20) σt,(st)δ,(st)=w(st)+∑p=1pαp,(st)(|εt−p|−γp,(st)εt−p,(st))δ,(st) +∑q=1qβq,(st)σt−q,(st)δ,(st)+∑h=1hξh,(st)ψ(τh,(st),Zt,(st)λh,(st),θh,(st)),

where, regimes are governed by unobservable Markov process. The model is closed as defining the conditional mean as in (12) and conditional variance of the form equation's (14)–(19) and (20) to augment the MS-ARMA-GARCH-MLP model with asymmetric power terms to obtain MS-ARMA-APGARCH-MLP. Note that, model nest several specifications. Equation (20) reduces to the MS-ARMA-GARCH-MLP model in (13) if the power term *δ* = 2 and *γ*_*p*,(*s*_*t*_)_ = 0. Similarly, the model nests MS-GJR-MLP if *δ* = 2 and 0 ≤ *γ*_*p*,(*s*_*t*_)_ ≤ 1 are imposed. The model may be shown as MSTGARCH-MLP model if *δ* = 1 and 0 ≤ *γ*_*p*,(*s*_*t*_)_ ≤ 1. Similarly, by applying a single regime restriction, *s*_*t*_ = *s* = 1, the quoted models reduce to their respective single regime variants, namely, the ARMA-APGARCH-MLP, ARMA-GARCH-MLP, ARMA-NGARCH-MLP, ARMA-GJRGARCH-MLP, and ARMA-GARCH-MLP models (for further discussion in NN-GARCH family models, see Bildirici and Ersin [61]).

#### 3.1.3. MS-ARMA-FIAPGARCH-MLP Model

Following the methodology discussed in the previous section, MS-ARMA-APGARCH-MLP model is augmented with neural network modeling architecture and that accounts for fractional integration to achieve long memory characteristics to obtain MS-ARMA-FIAPGARCH-MLP. Following the MS-ARMA-FIAPGARCH represented in (11), the MLP type neural network augmented MS-ARMA-FIAPGARCH-MLP model representation is achieved:

(21) (1−β(st)L)σt,(st)δ(st) =w(st)+((1−β(st)L)−(1−ϕ(st)L)(1−L)d(st))  ×(|εt−1,(st)|−γ(st)εt−1,(st))δ(st)  +∑h=1hξh,(st)ψ(τh,(st),Zt,(st)λh,(st),θh,(st)),

where, *h* are neurons defined with sigmoid type logistic functions, *i* = 1,…, *m* regime states governed by unobservable variable following Markov process. Equation (21) defines the MS-ARMA-FIAPGARCH-MLP model, the fractionally integration variant of the MSAGARCH-MLP model modified with the ANN, and the logistic activation function, *ψ*(*τ*_*h*,(*s*_*t*_)_, *Z*_*t*,(*s*_*t*_)_ *λ*_*h*,(*s*_*t*_)_, *θ*_*h*,(*s*_*t*_)_) defined as in (15). Bildirici and Ersin [61] proposes a class of NN-GARCH models including the NN-APGARCH. Similarly, the MS-ARMA-FIAPGARCH-MLP model reduces to the MS-FIGARCH-MLP model for restrictions on the power term *δ*_(*s*_*t*_)_ = 2 and *γ*_(*s*_*t*_)_ = 0. Further, the model reduces to MS-FINGARCH-MLP model for *γ*_(*s*_*t*_)_ = 0 and to the MS-FI-GJRGARCH-MLP model if *δ*_(*s*_*t*_)_ = 2 and *γ*_(*s*_*t*_)_ is restricted to be in the range of 0 ≤ *γ*_(*s*_*t*_)_ ≤ 1. The model reduces to MS-TGARCH-MLP model if *δ*_(*s*_*t*_)_ = 1 in addition to the 0 ≤ *γ*_(*s*_*t*_)_ ≤ 1 restriction. On the contrary, if single regime restriction is imposed, models discussed above, namely, MS-ARMA-FIAPGARCH-MLP, MSFIGARCH-NN, MSFIGARCH-NN, MSFINGARCH-MLP, MSFIGJRGARCH-MLP, and MSFITGARCH-MLP models reduce to NN-FIAPGARCH, NN-FIGARCH, NN-FIGARCH, NN-FINGARCH, NN-FIGJRGARCH, and NN-FITGARCH models, which are single regime neural network augmented GARCH family models of the form Bildirici and Ersin [61] that do not possess Markov switching type asymmetry (Bildirici and Ersin [61]). The model also nests model variants that do not possess long memory characteristics. By imposing *d*_(*s*_*t*_)_ = 0 to the fractional integration parameter which may take different values under *i* = 1,2,…, *m* different regimes, the model in (21) reduces to MS-ARMA-APGARCH-MLP model, the short memory model variant. In addition to the restrictions applied above, application of *d*_(*s*_*t*_)_ = 0 results in models without long memory characteristics: MS-ARMA-FIAPGARCH-MLP, MS-ARMA-GARCH-MLP, MS-ARMA-GARCH-MLP, MSNGARCH-MLP, MS-GJR-GARCH-MLP, and MSTGARCH-MLP.

For a typical example, consider a MS-ARMA-FIAPGARCH-MLP model representation with two regimes:

(22) (1−β(1)L)σt,(1)δ(1)=w(1)+((1−β(1)L)−(1−ϕ(1)L)(1−L)d(1)) ×(|εt−1|−γ(1)εt−1)δ(1)+∑h=1hξh,(1)ψ(τh,(1),Zt,(1)λh,(1),θh,(1)),(1−β(2)L)σt,(2)δ(2)=w(2)+((1−β(2)L)−(1−ϕ(2)L)(1−L)d(2)) ×(|εt−1|−γ(2)εt−1)δ(2)+∑h=1hξh,(2)ψ(τh,(2),Zt,(2)λh,(2),θh,(2)).

Following the division of regression space into two subspaces with Markov switching, the model allows two different asymmetric power terms, *δ*_(1)_ and *δ*_(2)_, and two different fractional differentiation parameters, *d*_(1)_ and *d*_(2)_; as a result, different long memory and asymmetric power structures are allowed in two distinguished regimes.

It is possible to show the model as a single regime NN-FIAPGARCH model if *i* = 1:

(23) (1−βL)σtδ=ω+((1−βL)−(1−ϕL)(1−L)d) ×(|εt−1|−γjεt−1)δ+∑h=1hξhψ(τh,Ztλh,θh).

Further, the model reduces to NN-FIGARCH if *i* = 1 and *δ*_(*s*_1_)_ = *δ* = 2 in the fashion of Bildirici and Ersin [61]:

(24) (1−βL)σt2=ω+((1−βL)−(1−ϕL)(1−L)d) ×(|εt−1|−γjεt−1)2+∑h=1hξhψ(τh,Ztλh,θh).

### 3.2. Radial Basis Function Model

*Radial Basis Functions *are one of the most commonly applied neural network models that aim at solving the interpolation problem encountered in nonlinear curve fitting.Liu and Zhang [120] utilized the* Radial Basis Function Neural Networks (RBF) *and Markov regime-switching regressionsto divide the regression space into two sub-spaces to overcome the difficulty in estimating the conditional volatility inherent in stock returns. Further, Santos et al. [121] developed a RBF-NN-GARCH model that benefit from the RBF type neural networks. Liu and Zhang [120] combined RBF neural network models with the Markov Switching model to merge Markov switching Neural Network model based on RBF models. RBF neural networks in their models are trained to generate both time series forecasts and certainty factors. Accordingly, RBF neural network is represented as a composition of three layers of nodes; first, the input layer that feeds the input data to each of the nodes in the second or hidden layer; the second layer that differs from other neural networks in that each node represents a data cluster which is centered at a particular point and has a given radius and in the third layer, consisting of one node.

#### 3.2.1. MS-ARMA-GARCH-RBF Model

MS-GARCH-RBF model is defined as

(25) σt,2(st)=w(st)+∑p=1pαp,(st)εt−p2,(st)+∑q=1qβq,(st)σt−q,(st)+∑h=1hξh,(st)ϕh,(st)(||Zt,(st)−μh,(st)||),

where *i* = 1,…, *m* regimes are governed by unobservable Markov process:

(26) $$\begin{array}{c} \overset{m}{\underset{i=1}{\sum }}\sigma_{t,(s_{t})}^{2}P\left(S_{t}=i\;|\;z_{t-1}^{}\right). \end{array}$$

A Gaussian basis function for the hidden units given as *ϕ*(*x*) for *x* = 1, 2,…, *X* where the activation function is defined as,

(27) $$\begin{array}{c} \phi \left(h,\left(s_{t}\right),Z_{t}\right)=\exp\left(\frac{-{||Z_{t,(s_{t})}-\mu_{h,(s_{t})}||}^{2}}{2\rho^{2}}\right). \end{array}$$

With *p* defining the width of each function. *Z*_*t*_ is a vector of lagged explanatory variables, *α* + *β* < 1 is essential to ensure stationarity. Networks of this type can generate any real-valued output, but in their applications where they have a priori knowledge of the range of the desired outputs, it is computationally more efficient to apply some nonlinear transfer function to the outputs to reflect that knowledge.

*P*(*S*_*t*_ = *i* | *z*_*t*−1_) is the filtered probability with the following representation:

(28) $$\begin{array}{c} \left(P\left(S_{t}=i\;|\;z_{t-1}^{}\right)\alpha f\left(P\left(\sigma_{t-1}\;|\;z_{t-1}^{},s_{t-1}=1\right)\right)\right). \end{array}$$

If *n*_*j*,*i*_ transition probability *P*(*s*_*t*_ = *i* | *s*_*t*−1_ = *j*) is accepted,

(29) $$\begin{array}{c} z_{t-d}=\frac{\left[\varepsilon_{t-d}-E\left(\varepsilon \right)\right]}{\sqrt{E\left(\varepsilon^{2}\right)}} \end{array}$$

*s* → max⁡{*p*, *q*} recursive procedure is started by constructing *P*(*z*_*s*_ = *i* | *z*_*s*−1_).

#### 3.2.2. MS-ARMA-APGARCH-RBF Model

Radial basis functions are three-layer neural network models with linear output functions and nonlinear activation functions defined as Gaussian functions in hidden layer utilized to the inputs in light of modeling a radial function of the distance between the inputs and calculated value in the hidden unit. The output unit produces a linear combination of the basis functions to provide a mapping between the input and output vectors:

(30) σt,(st)δ,(st)=w(st)+∑p=1pαp,(st)(|εt−j|−γp,(st)εt−p,(st))δ,(st) +∑q=1qβq,(st)σt−q,(st)δ,(st) +∑h=1hξh,(st)ϕh,(st)(||Zt,(st)−μh,(st)||),

where, *i* = 1,…, *m* regime model and regimes are governed by unobservable Markov process. Equations (26)–(29) with (30) define the MS-ARMA-APGARCH-RBF model. Similar to the MS-ARMA-APGARCH-MLP model, the MS-ARMA-APGARCH-RBF model nests several models. Equation (30) reduces to the MS-ARMA-GARCH-RBF model if the power term *δ* = 2 and *γ*_*p*,(*s*_*t*_)_ = 0, to the MSGARCH-RBF model for *γ*_*p*,(*s*_*t*_)_ = 0, and to the MSGJRGARCH-RBF model if *δ* = 2 and 0 ≤ *γ*_*p*,(*s*_*t*_)_ ≤ 1 restrictions are allowed. The model may be shown as MSTGARCH-RBF model if *δ* = 1 and 0 ≤ *γ*_*p*,(*s*_*t*_)_ ≤ 1. Further, single regime models, namely, NN-APGARCH, NN-GARCH, NN-GARCH, NN-NGARCH, NN-GJRGARCH, and NN-TGARCH models, may be obtained if *t* = 1 (for further discussion in NN-GARCH family models, see Bildirici and Ersin [61]).

#### 3.2.3. MS-ARMA-FIAPGARCH-RBF Model

MS-FIAPGARCH-RBF model is defined as

(31) (1−β(st)L)σt,(st)δ(st)=w(st)+((1−β(st)L)−(1−ϕ(st)L)(1−L)d(st)) ×(|εt−1,(st)|−γ(st)εt−1,(st))δ(st) +∑h=1hξh,(st)ϕh,(st)(||Zt,(st)−μh,(st)||),

where, *h* are neurons defined with Gaussian functions. The MS-ARMA-FIAPGARCH-RBF model is a variant of the MSAGARCH-RBF model with fractional integration augmented with ANN architecture. Similarly, the MS-ARMA-FIAPGARCH-RBF model reduces to the MS-ARMA-FIGARCH-RBF model with restrictions on the power term *δ*_(*s*_*t*_)_ = 2 and *γ*_(*s*_*t*_)_ = 0. The model nests MSFINGARCH-RBF model for *γ*_(*s*_*t*_)_ = 0, and MSFIGJRGARCH-RBF model if *δ*_(*s*_*t*_)_ = 2 and *γ*_(*s*_*t*_)_ varies between 0 ≤ *γ*_(*s*_*t*_)_ ≤ 1. Further, the model may be shown as MSTGARCH-RBF model if *δ*_(*s*_*t*_)_ = 1 and 0 ≤ *γ*_(*s*_*t*_)_ ≤ 1. With single regime restriction *i* = 1, discussed models reduce to NN-FIAPGARCH, NN-FIGARCH, NN-FIGARCH, NN-FINGARCH, NN-FIGJRGARCH, and NN-FITGARCH models, which do not possess Markov switching type asymmetry. To obtain the model with short memory characteristics, *d*_(·)_ = 0 restriction on fractional integration parameters should be imposed and the model reduces to MSAPGARCH-RBF model, the short memory model variant. Additionally, by applying *d*_(·)_ = 0 with the restrictions discussed above, models without long memory characteristics: MSFIAPGARCH-RBF, MSGARCH-RBF, MSGARCH-RBF, MSNGARCH-RBF, MSGJRGARCH-RBF, and MSTGARCH-RBF models could be obtained.

### 3.3. Recurrent Neural Network MS-GARCH Models

The RNN model includes the feed-forward system; however, it distinguishes itself from standard feed-forward network models in the activation characteristics within the layers. The activations are allowed to provide a feedback to units within the same or preceding layer(s). This forms an internal memory system that enables a RNN to construct sensitive internal representations in response to temporal features found within a data set.

The Jordan [122] and Elman's [123] networks are simple recurrent networks to obtain forecasts: Jordan and Elman networks extend the multilayer perceptron with context units, which are processing elements (PEs) that remember past activity. Context units provide the network with the ability to extract temporal information from the data. The RNN model employs back propagation-through-time, an efficient gradient-descent learning algorithm for recurrent networks. It was used as a standard variant of cross-validation referred to as the leave-one-out method and as a stopping criterion suitable for estimation problems with sparse data and so it is identified the onset of overfitting during training. The RNN was functionally equivalent to a nonlinear regression model used for time-series forecasting (Zhang et al. [124]; Binner et al. [125]). Tiňo et al. [126] merged the RNN and GARCH models.

#### 3.3.1. MS-ARMA-GARCH-RNN Models

The model is defined as

(32) σt,2(st)=w(st)+∑p=1pαp,(st)εt−p2,(st)+∑q=1qβq,(st)σt−q,(st)+∑h=1hξh,(st)πh,(st)(wk,h,(st)θt−k+θk,h,(st)).

Similar to the models above, (32) is shown for *i* = 1,…, *m* regimes which are governed by unobservable Markov process. Activation function is taken as the logistic function.

#### 3.3.2. MS-ARMA-APGARCH-RNN

Markov switching APGARCH Recurrent Neural Network Model is represented as

(33) σt,(st)δ,(st)=w(st)+∑p=1pαp,(st)(|εt−j|−γp,(st)εt−p,(st))δ,(st)+∑q=1qβq,(st)σt−q,(st)δ,(st)+∑h=1hξh,(st)Π(θk,h,(st)χt−k,h,(st)+θk,h,(st))

*i* = 1,…, *m* regimes are governed by unobservable Markov process. *θ*_*k*,*h*,(*s*_*t*_)_ is the weights of connection from pre to postsynaptic nodes, Π(*x*) is a logistic sigmoid function of the form given in (15), *χ*_*t*−*k*,*h*,(*s*_*t*_)_ is a variable vector corresponding to the activations of postsynaptic nodes, the output vector of the hidden units, and *θ*_*k*,*h*,(*s*_*t*_)_ are the bias parameters of the presynaptic nodes and *ξ*_*i*,(*s*_*t*_)_ are the weights of each hidden unit for *h* hidden neurons, *i* = 1,…, *h*. The parameters are estimated by minimizing the sum of the squared-error loss: $\min\lambda ={\sum_{t-1}^{T}\left[\sigma_{t}\;-\;{\hat{\sigma }}_{t}\right]}^{2}$. The model is estimated by recurrent back-propagation algorithm and by the recurrent Newton algorithm. By imposing several restrictions similar to the MS-ARMA-APGARCH-RBF model, several representations are shown under certain restrictions. Equation (33) reduces to MS-ARMA-GARCH-RNN model with *δ* = 2 and *γ*_*p*,(*s*_*t*_)_ = 0, to the MSGARCH-RNN model for *γ*_*p*,(*s*_*t*_)_ = 0, and to the MSGJRGARCH-RNN model if *δ* = 2 and 0 ≤ *γ*_*p*,(*s*_*t*_)_ ≤ 1 restrictions are imposed. MSTGARCH-RNN model is obtained if *δ* = 1 and 0 ≤ *γ*_*p*,(*s*_*t*_)_ ≤ 1. In addition to the restrictions above, if the single regime restriction *i* = 1 is implied, the model given in Equation (33) reduces to their single regime variants; namely, the APGARCH-RNN, GARCH-RNN, GJRGARCH-RNN, and TGARCH-RNN models, respectively.

#### 3.3.3. MS-ARMA-FIAPGARCH-RNN

Markov Switching Fractionally Integrated APGARCH Recurrent Neural Network Model is defined as

(34) (1−β(st)L)σt,(st)δ(st) =w(st)+((1−β(st)L)−(1−ϕ(st)L)(1−L)d(st))  ×(|εt−1,(st)|−γ(st)εt−1,(st))δ(st)  +∑h=1hξh,(st)Π(θk,h,(st)χt−k,h,(st)+θk,h,(st)),

where, *h* are neurons defined as sigmoid type logistic functions and *i* = 1,…, *m* regime states the following Markov process. The MS-ARMA-FIAPGARCH-RNN model is the fractionally integrated variant of the MS-ARMA-APGARCH-RNN model. The MS-ARMA-FIAPGARCH-RNN model reduces to the MS-ARMA-FIGARCH-RNN model with restrictions on the power term *δ*_(*s*_*t*_)_ = 2 and *γ*_(*s*_*t*_)_ = 0. Further, the model reduces to MSFINGARCH-RNN model for *γ*_(*s*_*t*_)_ = 0, to the MSFIGJRGARCH-RNN model if *δ*_(*s*_*t*_)_ = 2 and 0 ≤ *γ*_(*s*_*t*_)_ ≤ 1. The model reduces to MSFITGARCH-RNN model for 0 ≤ *γ*_(*s*_*t*_)_ ≤ 1 and *δ*_(*s*_*t*_)_ = 1. Single regime restriction *i* = 1 leads to the NN-FIAPGARCH-RNN, NN-FIGARCH-RNN, NN-FIGARCH-RNN, NN-FINGARCH-RNN, NN-FIGJRGARCH-RNN, and NN-FITGARCH-RNN models without Markov switching.

## 4. Data and Econometric Results

### 4.1. The Data

In order to test forecasting performance of the abovementioned models, stock return in Turkey is calculated by using the daily closing prices of Istanbul Stock Index ISE 100 covering the 07.12.1986–13.12.2010 period corresponding to 5852 observations. To obtain return series, the data is calculated as follows: *y*_*t*_ = ln⁡(*P*_*t*_/*P*_*t*−1_), where ln⁡(·) is the natural logarithms, *P*_*t*_ is the ISE 100 index, and *y*_*t*_ is taken as a measure of stock returns. In the process of training the models, the sample is divided between training, test, and out-of-sample subsamples with the percentages of 80%, 10%, and 10%. Further, we took the sample size for the training sample as the first 4680 observations, whereas the sample sizes for the test and out-of-sample samples are 585 and 587. The statistics of daily returns calculated from ISE 100 Index are given in Table 1.

In order to provide out-of-sample forecasts of the ISE100 daily returns, two competing nonlinear model structures are used, the univariate Markov switching model and Neural Network Models, MLP, RBF, and RNN. In order to assess the predictability of models, models are compared for their out-of-sample forecasting performance. Firstly, by calculating RMSE and MSE error criteria, the forecast comparisons are obtained.

### 4.2. Econometric Results

At the first stage, selected GARCH family models taken as baseline models are estimated for evaluation purposes. Results are given in Table 2. Included models have different characteristics to be evaluated, namely, fractional integration, asymmetric power, and fractionally integrated asymmetric power models, namely, GARCH, APGARCH, FIGARCH, and FIAPGARCH models. Random walk (RW) model is estimated for comparison purpose. Furthermore, the models given in Table 2 will provide basis for nonlinear models to be estimated.

It is observed that, though all volatility models perform better than the RW model in light of Log Likelihood criteria, as we move from the GARCH model to asymmetric power GARCH (APGARCH) model, the fit of the models improve accordingly. The sum of ARCH and GARCH parameters is calculated as 0.987 and less than 1. The results for the APGARCH model show that the calculated power term is 1.35 and the asymmetry is present. Further, similar to the findings of McKenzie and Mitchell [127], it is observed that the addition of the leverage and power terms improves generalization power and thus show that squared power term may not necessarily be optimal as Ding et al. [19] study suggested.

In Table 3, transition matrix and the MS model were estimated. The standard deviation takes the values of 0.05287 and 0.014572 for regime 1 and regime 2. It lasts approximately 75.87 months in regime 1 and 107.61 months in regime 2. By using maximum likelihood approach, MS-GARCH models are tested by assuming that the error terms follow student-*t* distribution with the help of BFGS algorithm. Number of regimes are taken as 2 and 3. GARCH effect in the residuals is tested and at 1% significance level, the hypothesis that there are no GARCH effects is rejected. Additionally, the normality in the residuals is tested with Jacque-Berra test, at 1% significance level, it is detected that the residuals are not normally distributed. As a result, MS-GARCH model is estimated under the *t* distribution assumption. In the MS-GARCH model, the transition probability results are calculated as Prob(*s*_*t*_ = 1 | *s*_*t*−1_ = 1) = 0.50 and Prob(*s*_*t*_ = 2 | *s*_*t*−1_ = 2) = 0.51 and show that the persistence is low in the MS-GARCH model.

Statistical inference regarding the empirical validity of two-regime switching process was carried out by using nonstandard LR tests [128]. The nonstandard LR test is statistically significant and this suggests that linearity is strongly rejected.

The volatility values tend to be calculated at higher values than they actually are in GARCH models. As the persistence coefficients obtained for the MS-GARCH, MS-PGARCH, and MS-APGARCH models are compared to those obtained for the GARCH models,; the persistence in the GARCH models are comparatively higher and for the GARCH models; a shock in volatility is persistent and shows continuing effect. This situation occurs as a result of omitting the importance of structural change in the ARCH process. In the MS-GARCH, MS-PGARCH, and MS-APGARCH models which take this situation into consideration, the value of persistence parameter decreases.

Power terms are reported comparatively lower for developed countries than the less developed countries. Haas et al. [109, 110] calculated three state RS-GARCH, RS-PGARCH, and RS-APGARCH models for the daily returns in NYSE and estimated the power terms for the RS-APGARCH model as 1.25, 1.09, and 1.08. For Turkey, Ural [129] estimated RS-APGARCH models for returns in ISE100 index in Turkey in addition to United Kingdom FTSE100, CAC40 in France and NIKKEI 225 indices in Japan and reported highest power estimates (1.84) compared to the power terms calculated as 1.26, 1.31, and 1.24 for FTSE100, NIKKEI 225, and CAC40. Further, power terms obtained for returns calculated for stock indices in many developing economies are calculated comparatively higher than those obtained for the various indices in developed countries. Ané and Ureche-Rangau [130] estimated single regime GARCH and APGARCH models in addition to RS-GARCH and RS-APGARCH models following Gray [92] model. Power terms in single regime APGARCH models were calculated for daily returns as 1.57 in Nikkei 225 Index, as 1.81 in Hang Seng Index, as 1.69 in Kuala Lumpur Composite Index, and as 2.41 in Singapore SES-ALL Index, whereas, for regime switching APGARCH models, power terms are calculated as 1.20 in regime 1 and 1.83 in regime 2 for Nikkei 225 Index, 2.16 in regime 1 and 2.31 in regime 2 for Heng Seng Index, 1.95 and 2.17 for regimes 1 and 2 in Singapore SES-ALL Index, and 1.71 and 2.25 in regimes 1 and 2 for the model calculated for returns in Kuala Lumpur Composite Index. Teletar and Binay [131] estimated APARCH models for Turkey and 10 national stock indices and noted that power terms reported for developing countries tend to be high and varying though those reported for the developed countries are estimated with low and close values. They estimated power terms for ISE100 index as 1.960 for 1987–2001 period and 1.48 for 1989–1996 period, whereas power terms for S&P, FTSE, NIKKEI, Hong Kong, NZSE40, DAX index of Germany, CAC40 of France, Singapore's SES, TSE, ALORAI, and MSCI indices as 1.21, 1.43, 1.22, 1.35, 1.37, 0.91, 1.17, 2.48, 1.45, 1.01, and 1.37, respectively.

On the other hand, though the improvement by shifting to modeling the conditional volatility with regime switching is noteworthy, the desired results are still not obtained, therefore, MS-GARCH models are extended with MLP, RBF, and RNN models and their modeling performances are tested.

#### 4.2.1. MS-ARMA-GARCH-Neural Networks Results

In the study, model estimation is gathered through utilizing back-propagation algorithm and the parameters are updated with respect to a quadratic loss function, whereas the weights are iteratively calculated with* weight decay* method to achieve the lowest error. Alternative methods include Genetic Algorithms [132–134] and 2nd order derivative based optimization algorithms such as Conjugate Gradient Descent, Quasi-Newton, Quick Propagation, Delta-Bar-Delta, and Levenberg-Marquardt, which are fast and effective algorithms but may be subject to overfitting (see [135–137]). In the study, we followed a two-step methodology. Firstly, all models were trained over a given training sample vis-à-vis checking for generalization accuracy in light of RMSE criteria in test sample. The approach is repeated for estimating each model for 100 times with different number of sigmoid activation functions in the hidden layer. Hence, to obtain parsimony in models, best model is further selected with respect to the AIC information criterion. For estimating NN-GARCH models with early stopping combined with algorithm corporation, readers are referred to Bildirici and Ersin [61].

The estimated models are reported in Table 4. For comparative purposes, MSE and RMSE values for training sample are given. Among the MS-ARMA-GARCH-NN models and for the training sample, the lowest RMSE value is achieved as 0.114 by the MS-ARMA-GARCH-RBF model, followed by MS-ARMA-GARCH-MLP model with a RMSE value of 0.186 as the second best model among the models with GARCH specifications noted at the first part of Table 4. Furthermore, the above mentioned models are followed by MS-ARMA-GARCH-HYBRID MLP model that deserves the 3rd place with RMSE = 0.187, though the value is almost equal to the value obtained for the MS-ARMA-GARCH-MLP model having the 2nd place. Further, MS-ARMA-GARCH-RNN, and MS-ARMA-GARCH-ELMAN RNN models take the 4th and 5th place among the GARCH type competing models in terms of in-sample accuracy only.

The Markov-switching models with asymmetric power terms in the conditional volatility, namely, the, MS-ARMA-APGARCH-NN models are reported in the second part of Table 4. The model group consists of MS-ARMA-APGARCH-RNN, MS-ARMA-APGARCH-RBF, MS-ARMA-APGARCH-ELMAN RNN, and MS-ARMA-APGARCH-MLP models. It is observed among the group that the lowest RMSE value is attained for the MS-ARMA-APGARCH-MLP model with a value of 0.16307 followed by MS-ARMA-APGARCH-RNN model. Further, MS-ARMA-APGARCH-ELMAN RNN and MS-ARMA-APGARCH-RNN models possess the 2nd and 3rd places with RMSE values equal to 0.1663 and 0.1776, respectively. Lastly, the lowest RMSE value (=0.2381) is attained for the MS-APGARCH-RBF model among the group.

Models in the third model group, namely, MS-ARMA-FIAPGARCH-RNN, MS-ARMA-FIAPGARCH-RBF, MS-ARMA-FIAPGARCH-RNN, MS-ARMA-FIAPGARCH-HYBRID MLP, and MS-ARMA-FIAPGARCH-MLP models are modified versions of MS-ARMA-APGARCH-NN models with fractional integration. According to the results obtained in Table 4, the lowest RMSE value is obtained for the MS-ARMA-FIAPGARCH-RNN model (RMSE = 0.1731) followed by the MS-ARMA-APGARCH-HYBRID MLP model (RMSE = 0.1762). The RMSE is calculated as 0.1767 for the MS-ARMA-FIAPGARCH-MLP model, which obtained the 3rd place, though it is observed that the models that deserved the first three places have very close RMSE values. Further, MS-ARMA-FIAPGARCH-ELMAN RNN, and MS-ARMA-FIAPGARCH-RBF models possess the 4th and 5th places with RMSE values of 0.1840 and 0.2144, with respect to the results obtained for the training sample.

The results obtained for the test sample and the hold-out sample are given in Tables 5 and 6. If an overlook is to be provided, it is observed that holding the training sample results on one side, the real improvement occurs for forecasting. When we move from MS-ARMA-GARCH-NN models (denoted as Model Group 1) towards MS-ARMA-APGARCH (denoted as Model Group 2) and fractionally integrated models of MS-ARMA-FIAPGARCH-NN (Model Group 3) models, though certain amount of in-sample forecast gain is achieved, the MSE and RMSE values are the lowest for test sample and, most importantly, for the out-of-sample forecasts.

To evaluate the test sample performance of the estimated models, MSE and RMSE error criteria are reported in Table 5. Accordingly, among the MS-ARMA-GARCH-NN models, the lowest RMSE value for the test sample is 0.1238 and is calculated for the MS-ARMA-GARCH-MLP model that is followed by the MS-ARMA-GARCH-RNN model with a RMSE of 0.1242. The MS-ARMA-GARCH-HYBRID MLP model takes the 3rd place in the test sample, similar to the results obtained for the training sample with RMSE = 0.1243, though the value is almost equal to the models taking the first and the second places. Compared with the first three models we mentioned with RMSE values that are achieved as low as around 0.124, MS-ARMA-GARCH-ELMAN RNN, and MS-ARMA-GARCH-RBF models take the 4th and 5th places with RMSE values equal to 0.1538 and 0.1546. The results show that, for the test sample, MS-ARMA-GARCH-MLP, MS-ARMA-GARCH-RNN, and MS-ARMA-GARCH-HYBRID MLP models have very similar results and well generalization capabilities, though the last 2 competing models also have promising results. Furthermore, as we evaluate the APGARCH type models, the generalization capabilities increase significantly. Among the MS-ARMA-APGARCH-NN models given in the table, MS-ARMA-APGARCH-HYBRID MLP model has the lowest RMSE (=0.00011539) followed by the MS-ARMA-APGARCH-MLP model (RMSE = 0.00011548). The third best model is the* MS-ARMA-APGARCH-ELMAN RNN model with *RMSE = 0.00011789 followed by the 4th and 5th models;* MS-ARMA-APGARCH-RNN* (RMSE = 0.0001276) and* MS-ARMA-APGARCH-RBF *(RMSE = 0.0001631), respectively. As discussed, models in the third group are MS-ARMA-FIAPGARCH-NN models. According to the results obtained in Table 5, the lowest RMSE values are obtained for the first three models: MS-ARMA-FIAPGARCH-RNN (RMSE = 0.001164), MS-ARMA-APGARCH-HYBRID MLP (RMSE = 0.0001177), and MS-ARMA-FIAPGARCH-MLP (RMSE = 0.0001178) models. Similar to the results in the test sample, the models that deserved the first three places have very close RMSE values. Similarly, MS-ARMA-FIAPGARCH-ELMAN RNN (RMSE = 0.000123) and MS-ARMA-FIAPGARCH-RBF (RMSE = 0.00016) models took the 4th and 5th places.

In Table 6, the models are compared for their respective out-of-sample forecast performances. At the first part of Table 6 MS-ARMA-GARCH-MLP has the lowest RMSE (=0.1238) in the MS-GARCH-NN group followed by MS-ARMA-GARCH-RNN (RMSE = 0.1242) and MS-ARMA-GARCH-HYBRID MLP (RMSE = 0.1243) models. Similar to the results obtained for the test sample, the MS-ARMA-APGARCH-NN and MS-ARMA-FIAPGARCH-NN specifications show significant improvement in light of MSE and RMSE criteria. Among the MS-ARMA-APGARCH-NN models, the lowest RMSE is achieved by MS-ARMA-APGARCH-HYBRID MLP (RMSE = 0.00011539) followed by the MS-ARMA-APGARCH-MLP (RMSE = 0.00011548) model. MS-ARMA-APGARCH-ELMAN RNN and MS-ARMA-APGARCH-RNN models took the 4th and 5th places in out-of-sample forecasting; however, though significant improvement is reported, MS-ARMA-APGARCH-RBF model took the 5th place among other MS-ARMA-APGARCH-NN models in the group. If MS-ARMA-FIAPGARCH-NN models are evaluated, the MS-ARMA-FIAPGARCH-RNN and MS-ARMA-FIAPGARCH-HYBRID MLP models are observed to take the 1st and 2nd places with respective RMSE values of 0.0001163 and 0.0001177. Further, the MS-ARMA-FIAPGARCH-MLP model is the 3rd with RMSE = 0.0001178 and the 4th and the 5th are MS-ARMA-FIAPGARCH-ELMAN RNN (RMSE = 0.0001233) and MS-FIAPGARCH-RBF (RMSE = 0.0001605) models. It is noteworthy that though all RMSE values are significantly low, RBF based model has become the last model among the MS-ARMA-FIAPGARCH-NN group members. Further, Diebold-Mariano equal forecast accuracy tests will be applied to evaluate the models.

To evaluate the forecast accuracy of the estimated models, Diebold-Mariano test results are reported in Table 7. The forecasting sample corresponds to the last 587 observations of ISE100 daily return series. For interpretation purposes, [r] and [c] given in brackets denote that the selected model is the row or the column model with respect to the *P* value given in parentheses for the calculated Diebold-Mariano test statistic.

Firstly, among the models in Group 1, the MS-ARMA-GARCH-RNN model is compared with the MS-ARMA-GARCH-RBF model in the first column. The calculated DM test statistic is −4.020 and significant at the 1% significance level, thus, the null hypothesis of equal forecast accuracy is rejected in favor of MS-ARMA-GARCH-RNN model. The MS-ARMA-GARCH-RBF model is compared with the MS-ARMA-GARCH-RNN model. The DM statistic is −3.176 and shows that at 5% significance level MS-ARMA-GARCH-RNN model is selected over MS-ARMA-GARCH-ELMAN RNN model. If MS-ARMA-GARCH-HYBRID MLP and MS-ARMA-GARCH-RNN models are compared, we fail to reject the null hypothesis of equal forecast accuracy. If MS-ARMA-GARCH-MLP and MS-ARMA-GARCH-RNN models are compared, DM test statistic is calculated as 0.574 with *P* value = 0.56, therefore, the null hypothesis of equal forecast accuracy cannot be rejected. The results show that MS-ARMA-GARCH-RNN model is preferred over MS-ARMA-GARCH-RBF and MS-ARMA-GARCH-ELMAN RNN models. If the MS-ARMA-GARCH-RBF model at the second row is analyzed, the null of equal forecast accuracy cannot be rejected between MS-ARMA-GARCH-RBF and MS-ARMA-GARCH-ELMAN RNN models, whereas MS-ARMA-GARCH-HYBRID MLP (DM = 4.011) and MS-ARMA-GARCH-MLP (DM = 4.142) models are selected over MS-ARMA-GARCH-RBF model. The equal forecast accuracy of MS-ARMA-GARCH-ELMAN RNN and MS-ARMA-GARCH-HYBRID MLP models is rejected and the alternative hypothesis that MS-ARMA-GARCH-HYBRID MLP model has better forecast accuracy is accepted at 1% significance level (DM = 3.309). The DM statistic calculated for MS-ARMA-GARCH-ELMAN RNN and MS-ARMA-GARCH-MLP models is 3.396 suggesting that MS-GARCH-MLP model is selected.

The asymmetric power GARCH architecture based models in Group 2 are evaluated at the second part of Table 7. MS-ARMA-APGARCH-RNN is compared with the other models at the first row. DM test statistic is calculated as −2.932 suggesting that the null of equal forecast accuracy between MS-ARMA-APGARCH-RNN and MS-ARMA-APGARCH-RBF is rejected in favor of MS-ARMA-APGARCH-RNN model. Further, the null hypotheses for the test of equal forecast accuracy between MS-ARMA-APGARCH-RNN and the other models are rejected and better forecast accuracy of MS-ARMA-APGARCH-ELMAN RNN, MS-ARMA-APGARCH-HYBRID MLP, and MS-ARMA-APGARCH-MLP are accepted at 1% significance level. Similar to the analysis conducted before, RBF based MS-ARMA-APGARCH-RBF model fail to have better forecast accuracy than the other models at the second row. MS-ARMA-APGARCH-HYBRID MLP model proved to have better forecast accuracy than all other models, whereas MS-ARMA-APGARCH-MLP model show to provide better forecast accuracy than all other models except for the MS-ARMA-APGARCH-HYBRID MLP model at 1% significance level.

MS-ARMA-FIAPGARCH-NN results are given in the third section of Table 7. The MS-ARMA-FIAPGARCH-RNN model is compared with the MS-ARMA-FIAPGARCH-RBF model in the first column. The calculated DM test statistic is −2.519 and suggests that the null hypothesis of equal forecast accuracy is rejected in favor of MS-ARMA-FIAPGARCH-RNN model. The DM statistic is −1.717 and shows that MS-ARMA-FIAPGARCH-RNN model is selected over MS-ARMA-FIAPGARCH-ELMAN RNN model only at 10% significance level. If we compare the MS-ARMA-FIAPGARCH-HYBRID MLP and MS-ARMA-GARCH-RNN models, we fail to reject the null hypothesis. On the other hand, MS-FIAPGARCH-HYBRID MLP is preferred over MS-ARMA-FIAPGARCH-ELMAN RNN at 5% significance level (DM = 2.154) and over MS-ARMA-FIAPGARCH-RBF model at 1% significance level (DM = 2.608). Accordingly, MS-ARMA-FIAPGARCH-RBF model fail to beat other models though RMSE values show significant improvement, the MS-ARMA-FIAPGARCH-RNN model is selected over the other models, whereas the MS-ARMA-FIAPGARCH-HYBRID MLP model is the second best model for the analyzed time series and analyzed sample.

## 5. Conclusions

In the study, a family of regime switching neural network augmented volatility models is analyzed to achieve an evaluation of forecast accuracy and an application to daily returns in an emerging market stock index presented. In this respect, GARCH neural network models, which allow the generalization of MS type RS-GARCH models to MS-ARMA-GARCH-NN models are incorporated with neural networks models based on Multilayer Perceptron (MLP), Radial Basis Function (RBF), Elman Recurrent NN (Elman RNN), Time Lag (delay) Recurrent NN (RNN), and Hybrid MLP models. Gray [92] RS-GARCH model is taken as the baseline modeling structure in the study. Gray [92] utilizes the Hamilton [67] Markov-switching approach, however the regime-switching is allowed in the conditional volatility processes of a time series. On the other hand, the study aimed at Hamilton [67] type regime-switching between GARCH-Neural Network approaches developed by Donaldson and Kamstra [118] and further extanded to a class of GARCH-NN models by Bildirici and Ersin [61]. The model within the family analyzed are MS-ARMA-GARCH-MLP, MS-ARMA-GARCH-RBF, MS-ARMA-GARCH-ElmanRNN, MS-ARMA-GARCH-RNN, and MS-ARMA-GARCH-Hybrid MLP which are further extended to account for asymmetric power terms based on the APGARCH architecture, MS-ARMA-APGARCH-MLP, MS-ARMA-APGARCH-RBF, MS-ARMA-APGARCH-ElmanRNN, MS-ARMA-APGARCH-RNN, and MS-ARMA-APGARCH-Hybrid MLP.

Further, by accounting for fractional integration (FI) in GARCH specification, the models are generalized as MS-ARMA-FIAPGARCH-MLP, MS-ARMA-FIAPGARCH-RBF, MS-ARMA-FIAPGARCH-ElmanRNN, MS-ARMA-FIAPGARCH-RNN, and MS-ARMA-FIAPGARCH-Hybrid MLP models that account for fractionally integrated models with asymmetric power transformations and generalized to neural network models with possibly improved forecast capabilities.

Models are evaluated with MSE and RMSE error criteria. To evaluate equal forecast accuracy, modified Diebold-Mariano tests are applied. It is observed that, holding the training sample results on one side, the real improvement occurs for the test sample and most importantly, for the out-of-sample. By evaluating MS-GARCH-NN models, though in-sample performance is noticeable, moving towards MS-ARMA-APGARCH-NN models and fractionally integrated models of MS-ARMA-FIAPGARCH-NN, significant improvement is noticed in light of the MSE and RMSE criteria and in terms of Diebold-Mariano equal forecast accuracy tests.

Among the models analyzed, asymmetric power modeling with fractional integration generalized to Time Lag Recurrent Neural Network architecture and Hybrid Multilayer Perceptron are shown to provide significant forecast and modeling capabilities. Thus, the models with regime switching further augmented with neural network modeling techniques promise significant achievements in return and volatility modeling and forecasting.

## Figures and Tables

**Table 1:** Daily returns in ISE 100 index, basic statistics.

Mean	Median	Mode	Minimum	Maximum	Standard deviation	Skewness	Kurtosis
0.001725	0.001421	0.0000001	−0.19979	0.217108	0.028795	0.071831	4.207437

**Table 2:** Baseline volatility models.

Baseline GARCH models, single regime								
(1) RW	C							log⁡L

	0.001873*** (0.000391)							12241.56

(2) GARCH	ARCH	GARCH	C					log⁡L

	0.1591259*** (0.0068625)	0.8280324*** (0.0056163)	0.0000206*** (1.80*e* − 06)					13174.66

(3) APGARCH	APARCH	APARCH_E	PGARCH	POWER	C			log⁡L

	0.2153934*** (0.0076925)	−0.0319876*** (0.0160775)	0.7801279*** (0.0074049)	1.354999*** (0.0707303)	0.0004055*** (0.0001074)			13111.96

(4) FIAPGARCH	ARCH (Phi1)	GARCH (Beta1)	D-FIGARCH	APARCH (Gamma1)	APARCH (Delta)	C (MEAN)	C (VAR.)	log⁡L

	0.250877*** (0.096563)	0.449631*** (0.10367)	0.417296*** (0.040388)	0.027382 (0.033565)	2.020946*** (0.096663)	0.001635*** (0.00028929)	8.054283 (6.6685)	13196.30

**Table 3:** MS-ARMA-GARCH models.

MS-ARMA-GARCH models										
(1) MS-ARMA-GARCH	Garch	Sigma	Arch	Constant	p_{0 | 0}	*P*_{1 | 1}			log⁡*L*	RMSE

Regime 1.	0.966483 (0.01307)***	0.000333727 (1.916*e* − 006)***	0.03351 (0.005)	6.23008*e* − 005 (1.360*e* − 005)***	0.500244	0.5102			385.09	0.458911
Regime 2.	0.436124 (0.01307)***	0.0004356 (1.231*e* − 005)***	0.56387 (0.0098)	6.29344*e* − 005 (1.161*e* − 005)***						

(2) MS-ARMA-APGARCH	Garch	sigma	Arch	Constant	Mean	*P*_{0 | 0}	*p*_{1 | 1}	Power	log⁡*L*	RMSE

Regime 1.	0.616759 (0.01307)***	0.000679791 (5.685*e* − 006)***	0.383241 (0.0102)	8.13394*e* − 005 (2.007*e* − 005)***	0.00021042	0.500227	0.50300	0.80456 (0.00546)	1756.5	0.42111
Regime 2.	0.791950 (0.01307)***	0.0012381 (3.451*e* − 004)***	0.20805 (0.0201)	8.20782*e* − 005 (1.835*e* − 005)***	2.83*E* − 03			0.60567 (0.0234)		

(3) MS-ARMA-FIAPGARCH	ARCH (Phi1)	GARCH (Beta1)	d-Figarch	APARCH (Gamma1)	APARCH (Delta)	Cst	*p*_{0 | 0}	*p*_{0 | 1}	log⁡*L*	RMSE

Regime 1.	0.277721 (0.00)	0.67848 (0.00)	0.2761233 (0.0266)	0.220157 (0.0106)	0.123656 (0.001)	0.00135 (0.001)	0.50212	0.510021	1877.9	0.4222066
Regime 2.	0.309385 (0.002)	0.680615 (0.0001)	0.181542 (0.00005)	0.21083 (0.0299)	0.1448 (0.0234)	0.00112 (0.00984)				

**Table 4:** Markov switching GARCH neural network models: training results.

	MSE	RMSE
Model Group 1: MS-ARMA-GARCH-neural network models		
MS-ARMA-GARCH-RNN	0.035103533	0.187359368 (4th)
MS-ARMA-GARCH-RBF	0.013009577	0.114059534 (1st)
MS-ARMA-GARCH-ELMAN RNN	0.051024351	0.225885704 (5th)
MS-ARMA-GARCH-HYBRID MLP	0.034996508	0.187073536 (3rd)
MS-ARMA-GARCH-MLP	0.034665716	0.186187315 (2nd)

Model Group 2: MS-ARMA-APGARCH-neural network models		
MS-ARMA-APGARCH-RNN	0.031530707	0.177568881 (4th)
MS-ARMA-APGARCH-RBF	0.056680761	0.238077218 (5th)
MS-ARMA-APGARCH-ELMAN RNN	0.027644691	0.166266929 (3rd)
MS-ARMA-APGARCH-HYBRID MLP	0.026504969	0.162803470 (1st)
MS-ARMA-APGARCH-MLP	0.026591934	0.163070336 (2nd)

Model Group 3: MS-ARMA-FIAPGARCH-neural network models		
MS-ARMA-FIAPGARCH-RNN	0.029951509	0.173065042 (1st)
MS-ARMA-ARMA-FIAPGARCH-RBF	0.045969206	0.214404305 (5th)
MS-ARMA-FIAPGARCH-ELMAN RNN	0.033859273	0.184008896 (4th)
MS-ARMA-FIAPGARCH-HYBRID MLP	0.031056323	0.176228044 (2nd)
MS-ARMA-FIAPGARCH-MLP	0.031221803	0.176696926 (3rd)

**Table 5:** Markov switching ARMA-GARCH neural network models: test sample results.

	MSE	RMSE
Model Group 1: MS-ARMA-GARCH-neural network models		
MS-ARMA-GARCH-RNN	0.015436917	0.124245392 (2nd)
MS-ARMA-GARCH-RBF	0.023914916	0.154644483 (5th)
MS-ARMA-GARCH-ELMAN RNN	0.023667136	0.153841268 (4th)
MS-ARMA-GARCH-HYBRID MLP	0.015461623	0.124344774 (3rd)
MS-ARMA-GARCH-MLP	0.015333378	0.123828017 (1st)

Model Group 2: MS-ARMA-APGARCH-neural network models		
MS-ARMA-APGARCH-RNN	0.00000001628449	0.00012761067957 (4th)
MS-ARMA-APGARCH-RBF	0.00000002662825	0.00016318165387 (5th)
MS-ARMA-APGARCH-ELMAN RNN	0.00000001389858	0.00011789225071 (3rd)
MS-ARMA-APGARCH-HYBRID MLP	0.00000001331575	0.00011539391162 (1st)
MS-ARMA-APGARCH-MLP	0.00000001333791	0.00011548986313 (2nd)

Model Group 3: MS-ARMA-FIAPGARCH-neural network models		
MS-ARMA-FIAPGARCH-RNN	0.00000001354729	0.00011639284333 (1st)
MS-ARMA-FIAPGARCH-RBF	0.00000002576468	0.00016051379977 (5th)
MS-ARMA-FIAPGARCH-ELMAN RNN	0.00000001521219	0.00012333770222 (4th)
MS-ARMA-FIAPGARCH-HYBRID MLP	0.00000001385396	0.00011770286713 (2nd)
MS-ARMA-FIAPGARCH-MLP	0.00000001389543	0.00011787886146 (3rd)

**Table 6:** Markov switching ARMA-GARCH neural network models: out of sample results.

	MSE	RMSE
Model Group 1: MS-ARMA-GARCH-neural network models		
MS-ARMA-GARCH	0.210599000	0.458911000 (6th)
MS-ARMA-GARCH-RNN	0.015436917	0.124245392 (2nd)
MS-ARMA-GARCH-RBF	0.023914916	0.154644483 (5th)
MS-ARMA-GARCH-ELMAN RNN	0.023667136	0.153841268 (4th)
MS-ARMA-GARCH-HYBRID MLP	0.015461623	0.124344774 (3rd)
MS-ARMA-GARCH-MLP	0.015333378	0.123828017 (1st)

Model Group 2: MS-ARMA-APGARCH-neural network models		
MS-ARMA-APGARCH	0.17740000000000	0.42111000000000 (6th)
MS-ARMA-APGARCH-RNN	0.00000001628449	0.00012761067957 (4th)
MS-ARMA-APGARCH-RBF	0.00000002662825	0.00016318165387 (5th)
MS-ARMA-APGARCH-ELMAN RNN	0.00000001389858	0.00011789225071 (3rd)
MS-ARMA-APGARCH-HYBRID MLP	0.00000001331575	0.00011539391162 (1st)
MS-ARMA-APGARCH-MLP	0.00000001333791	0.00011548986313 (2nd)

Model Group 3: MS-ARMA-FIAPGARCH-neural network models		
MS-ARMA-FIAPGARCH	0.17814000000000	0.42220660000000 (6th)
MS-ARMA-FIAPGARCH-RNN	0.00000001354729	0.00011639284333 (1st)
MS-ARMA-FIAPGARCH-RBF	0.00000002576468	0.00016051379977 (5th)
MS-ARMA-FIAPGARCH-ELMAN RNN	0.00000001521219	0.00012333770222 (4th)
MS-ARMA-FIAPGARCH-HYBRID MLP	0.00000001385396	0.00011770286713 (2nd)
MS-ARMA-FIAPGARCH-MLP	0.00000001389543	0.00011787886146 (3rd)

**Table 7:** Diebold Mariano equal forecast accuracy test results, out of sample.

Model Group 1: MS-ARMA-GARCH-neural network models					
	MS-ARMA- GARCH- RNN	MS-ARMA- GARCH- RBF	MS-ARMA- GARCH- ELMAN RNN	MS-ARMA- GARCH- HYBRID MLP	MS-ARMA- GARCH- MLP
MS-ARMA-GARCH- RNN	—	−4.020*** (0.000) [r]	−3.176** (0.002) [r]	−0.645 (0.518) [r]	0.574 (0.566) [c]
MS-ARMA-GARCH- RBF		—	0.585 (0.558) [c]	4.011*** (0.000) [c]	4.142*** (0.000) [c]
MS-ARMA-GARCH-ELMAN RNN			—	3.309*** (0.001) [c]	3.396*** (0.001) [c]
MS-ARMA-GARCH-HYBRID MLP				—	3.355*** (0.01) [c]
MS-ARMA-GARCH- MLP					—

Model Group 2: MS-ARMA-APGARCH-neural network models					
	MS-ARMA- APGARCH- RNN	MS-ARMA- APGARCH- RBF	MS-ARMA- APGARCH- ELMAN RNN	MS-ARMA- APGARCH- HYBRID MLP	MS-ARMA- APGARCH- MLP

MS-ARMA-APGARCH-RNN	—	−2.932*** (0.003) [r]	3.767*** (0.000) [c]	4.888*** (0.000) [c]	4.805*** (0.000) [c]
MS-ARMA-APGARCH- RBF		—	3.188*** (0.001) [c]	3.251*** (0.001) [c]	3.255*** (0.001) [c]
MS-ARMA-APGARCH-ELMAN RNN			—	2.797*** (0.005) [c]	2.736*** (0.006) [c]
MS-ARMA-APGARCH-HYBRID MLP				—	−1.835** (0.066) [r]
MS-ARMA-APGARCH-MLP					—

Model Group 3: MS-ARMA-FIAPGARCH-neural network models					
	MS-ARMA-FIAPGARCH- RNN	MS-ARMA-FIAPGARCH- RBF	MS-ARMA- FIAPGARCH- ELMAN RNN	MS-ARMA-FIAPGARCH- HYBRID MLP	MS-ARMA-FIAPGARCH- MLP

MS-ARMA-FIAPGARCH-RNN	—	−2.519** (0.011) [r]	−1.717* (0.086) [r]	−0.588 (0.556) [r]	−0.836 (0.403) [r]
MS-ARMA-FIAPGARCH-RBF		—	2.214** (0.027) [c]	2.608*** (0.009) [c]	2.526** (0.012) [c]
MS-ARMA-FIAPGARCH-ELMAN RNN			—	2.154** (0.031) [c]	−2.214** (0.026) [r]
MS-ARMA-FIAPGARCH-HYBRID MLP				—	−0.716 (0.473) [r]
MS-ARMA-FIAPGARCH-MLP					—

Notes: Statistical significances of the relevant tests are denoted with ***show statistical significance at 1% significance level; while ^∗∗, and ∗^show significance at 5% and 10%, respectively. D-M test allows for reporting the selected model. Accordingly, [r] shows that the model reported in the “row” is selected over the model in the column. Similarly, [c] stands for the column model being accepted over the row model.
